# Miltefosine implementation for treatment of cutaneous leishmaniasis: access indicators in the state of Minas Gerais, 2021-2024

**DOI:** 10.1590/S2237-96222025v34e20240690.en

**Published:** 2025-06-20

**Authors:** Sarah Nascimento Silva, Mell Ferreira Saliba, Laís Raquel Ribeiro, Gláucia Cota

**Affiliations:** 1 Núcleo de Avaliação de Tecnologias em Saúde, Instituto René Rachou, Fundação Oswaldo Cruz, Belo Horizonte, MG, Brazil

**Keywords:** Leishmaniosis, Access to Essential Medicines and Health Technologies, Implementation Science, Brazilian National Health System, Cross-Sectional Studies, Leishmaniosis, Acceso a Medicamentos Esenciales y Tecnologías Sanitarias, Ciencia de la Implementación, Sistema Único de Salud, Estudios Transversales

## Abstract

**Objective:**

To describe and evaluate the initial indicators of the pilot implementation of miltefosine in Minas Gerais for the treatment of cutaneous leishmaniasis.

**Methods:**

This is a descriptive, cross-sectional study based on regulatory documents and secondary administrative data generated during the dispensing of miltefosine in Minas Gerais between May 2021 and April 2024. Indicators related to access and operability of the strategy implemented were calculated for the steps involved in approving the request for use and dispensing of the drug.

**Results:**

The authorization rate was 97.5% for 281 valid requests. The outpatient clinic of the René Rachou Institute, a reference center for leishmaniasis in Minas Gerais, was responsible for the largest number of requests during the period. The health macro-regions Centre and North concentrated the largest number of requests (48.9% and 33.6%), which coincided with the regions with the highest number of reported cases. Median time for analyzing requests and for the person to access the medicine was 1.0 (0-76) and 10.0 (0-340) days, with significant variation between the health macro-regions. One hundred and nineteen people were treated in a municipality other than their place of residence. Of these, 49 needed to travel to another health macro-region. The miltefosine diffusion rate was 11.3% for the three-year period monitored, with significant variation between the macro-regions.

**Conclusion:**

The indicators confirmed that miltefosine has been implemented, but revealed low diffusion and regional disparities. Barriers need to be identified and strategies developed to expand equitable access to new technologies.

## Resumen

**Objetivo:**

Describir y evaluar los primeros indicadores de la implementación piloto de miltefosina en Minas Gerais para el tratamiento de la leishmaniasis cutánea.

**Métodos:**

Se trata de un estudio descriptivo y transversal, basado en documentos normativos y datos administrativos secundarios, generados durante la dispensación de miltefosina en Minas Gerais entre mayo de 2021 y abril de 2024. Se calcularon indicadores relacionados con el acceso y la operatividad de la estrategia implementada para los pasos involucrados en la aprobación de la solicitud de uso y dispensación del medicamento.

**Resultados:**

La tasa de aprobación fue del 97,5% para 281 solicitudes válidas. El ambulatorio del Instituto René Rachou, centro de referencia en leishmaniasis en Minas Gerais, fue responsable por el mayor número de solicitudes en el período. Las macrorregiones sanitarias Centro y Norte concentraron el mayor número de solicitudes (48,9% y 33,6%), lo que coincidió con las regiones con mayor número de casos reportados. Las medianas de tiempo para el análisis de las solicitudes y el acceso de la persona al medicamento fueron de 1,0 (0-76) y 10,0 (0-340) días, con gran variación entre las macrorregiones de salud. Fueron atendidas 119 personas en municipio distinto de su residencia. De ellos, 49 necesitaron trasladarse a otra macrorregión sanitaria. La tasa de difusión del fármaco fue del 11,3% durante los tres años de seguimiento, con una gran variación entre las macrorregiones.

**Conclusión:**

Los indicadores confirmaron la implementación de miltefosina, pero revelaron baja difusión y disparidades regionales. Por lo tanto, es necesario identificar barreras y delinear estrategias para ampliar el acceso equitativo a las nuevas tecnologías.

**Ethical aspects**

This research respected ethical principles, having obtained the following approval data::

Research Ethics Committee: Instituto René Rachou

Opinion number: 5,238,337

Approval date: 20/12/2021

Certificate of Submission for Ethical Appraisal: 45481421.4.0000.5091

Informed Consent Form: Obtained from all participants prior to data collection.

## Introduction

Cutaneous leishmaniasis is an infectious, non-contagious disease caused by several species of the genus Leishmania and transmitted by mosquito bites, predominantly in tropical regions (1). With wide global distribution, it mainly affects neglected populations in underdeveloped areas, which causes considerable morbidity and socioeconomic impact (2). Brazil was among the eight countries accounting for 85.0% of global cases, with an annual average of 16,000 cases recorded between 2018 and 2022 (3). The disease represents a challenge from a public policy perspective, as it requires technical, operational, and political effort. There is difficulty in intervening in the transmission cycle, which is associated with environmental interaction and wide geographic distribution in the Americas (1,4).

Access to cutaneous leishmaniasis treatment is one of the strategies for controlling the disease, but it is limited by difficulties in diagnosis and the limited number of therapeutic options, which include parenteral treatments, administered for a long time and associated with considerable toxicity (1). Miltefosine is the first oral medicine for the treatment of visceral and cutaneous leishmaniasis, and its availability represents significant progress in terms of decentralizing access by allowing self-administration at home (5,6). Miltefosine was incorporated into the Brazilian National Health System (*Sistema Único de Saúde* - SUS) in 2018 and, after a long regulatory process (7) and international purchasing, it began to be made available by the Ministry of Health to state Health Departments in 2021, via the Strategic Component of Pharmaceutical Assistance (8). The teratogenic potential of miltefosine has required strict control of its use and a team prepared to monitor users, with a special focus on women of childbearing age (9).

Implementing medicine recently incorporated into the SUS brings several challenges that go beyond its availability in the health service. Overcoming these challenges requires understanding the obstacles to its provision (10), resistance or lack of knowledge about its availability, and adoption of strategies that consolidate its use in the clinical practice of health services. Although medicine incorporation processes are well defined in the SUS (11), for health service managers implementation processes have gaps in terms of federal guidelines and guidance on executing them (12). Indicators related to access to medicines in the health system constitute fundamental data for understanding the capillarity with which their being provided was made, this being one of the essential factors for effective implementation (13).

Once the Ministry of Health began distributing miltefosine, each state had autonomy to define its own distribution strategy (14). In Minas Gerais, a region where cutaneous leishmaniasis is endemic, the state Health Department entered into a technical support partnership with the René Rachou Institute Leishmaniasis Reference Center for the design and execution of the miltefosine pilot implementation plan (15). This experience represented an opportunity to develop a systematic strategy to guide and monitor the implementation of health technologies in Brazil. The objective of this study was to describe and evaluate the initial indicators of the miltefosine pilot implementation strategy in Minas Gerais.

## Methods

### Design and setting

This is a descriptive and cross-sectional study based on secondary sources of information. It describes the experience of the pilot implementation of miltefosine in Minas Gerais and analysis of access indicators, between May 2021 and April 2024, totaling 36 months.

### Participants and variables 

The participants were SUS users diagnosed with cutaneous leishmaniasis for whom treatment with miltefosine was prescribed. The following sociodemographic variables were analyzed: sex (female, male), age, place of residence (municipality), education (illiterate, elementary, high school, higher education) and race/skin color. Clinical variables were analyzed regarding the form of the disease (cutaneous, mucosal), type of case (new, recurrence), date of diagnosis and childbearing potential (at risk, not at risk). Variables related to the processes of requesting miltefosine were described by the following variables: place of residence and service location (municipality and health macro-region of Minas Gerais defined according to the state’s regionalization master plan), distance traveled, date of request, date of issuance of opinion and dates of dispensing and recording on the system. The following measures were chosen as indicators of access:

1. **Rate of authorization of miltefosine requests**: ratio between the number of authorized requests in relation to the total number of requests filed with the state Health Department that were considered valid (excluding duplicates), multiplied by 100.
2. **Rate of women of childbearing age using miltefosine**: ratio between the number of women of childbearing age and the total number of people treated with miltefosine, multiplied by 100. Women of childbearing age were defined as those between 12 and 55 years old, excluding those who had undergone a definitive sterilization procedure, or women in menopause confirmed for at least two years.
3. **Request analysis time**: interval in days between the date on which the email with the request for miltefosine was received at the state Health Department (protocol) and the date of issuance of the final opinion. The Reference Service is excluded from this analysis, as this step is not part of its procedures.
4. **Access to medicine time**: interval in days between the date on which the email with the request for miltefosine was received at the state Health Department (protocol) and the date on which the medicine was dispensed to the service user at the designated pharmacy.
5. **Time between diagnosis and dispensing of medicine**: interval in days between the date of diagnosis of cutaneous leishmaniasis and the day on which the medicine was dispensed to the service user. Calculated only for new cases of the disease, it reflects the total time taken to access to treatment after confirmation of diagnosis of cutaneous leishmaniasis.
6. **Dispensing recording rate** (**first stage**): ratio between the number of miltefosine dispensings recorded on the dispensing system and the number of authorizations for dispensing miltefosine given by the state Health Department in the first stage of dispensing the drug, multiplied by 100. Reflects the adequacy of the dispensing recording process, since recording on the dispensing system is mandatory for miltefosine.
7. **Degree of diffusion**: ratio between the number of authorized requests for miltefosine and the total number of cases of cutaneous leishmaniasis diagnosed in the state for the period published on the TabNet health platform until September 9, 2024. This is a measure of the penetration, that is, the integration of the intervention into the practice.

### Data sources and measurement 

Administrative data from documents related to the request and prescription of miltefosine form the study data source: prescription, diagnostic test report, medical report, medicine request form, cutaneous leishmaniasis case reporting form and the Notifiable Health Conditions Information System form. We also analyzed other documents generated within the scope of the strategic component of pharmaceutical assistance during this period, related to management of miltefosine requests, such as spreadsheets containing requests, communication emails for analysis of requests and the miltefosine accounting record based on reports from the Integrated Pharmaceutical Assistance Management System.

### Bias control and study size 

Miltefosine request data were compiled into a database by pairing information on its request, authorization or refusal reply, and dispensing dates. The sample for this study comprised all request records generated during the period analyzed. After the database was cleaned by removing duplicates, the main database was pseudo-anonymized by two independent researchers, generating a new database for the analyses.

### Statistical methods 

The population that had access to miltefosine was characterized based on the description of measures of central tendency and dispersion (lower-upper) for sociodemographic and clinical variables using the Statistical Package for the Social Sciences, version 23. Measures related to access and operability of the miltefosine implementation strategy were calculated according to the indicators defined. The results were presented overall and stratified by health macro-regions. Analysis of travel was performed for all users, identifying the distance traveled between the place of residence and the service that dispended the medicine. With regard to the service with the largest number of external users, the municipalities were georeferenced on the map of Minas Gerais based on the location of the municipalities of residence, using GeoDa software, version 1.22.0.4. This study adopted the REporting of studies Conducted using Observational Routinely-collected health Data (16) and the Strengthening the Reporting of Observational Studies in Epidemiology (17) statement checklists.

## Results

### Description of the implementation strategy

The implementation strategy adopted was structured in stages, inspired by the Deming Cycle process management and control tool, with its Plan-Do-Check-Act stages. These were adapted to comprise four sets of actions called “map, prepare, pilot and monitor”, giving rise to the generic approach to guide implementation in healthcare, which was given the name ImplementaSUS. 

After analyzing the context in which the new technology would be used and the requirements already established for its use, the authorization flow for use and distribution to be adopted in the state of Minas Gerais was defined, as well as the set of minimum documents that should support prescription of treatment with miltefosine, and the actions to monitor these processes. The Reference Center adopted a different flow with the issuance of an internal authorization report and immediate dispensing of the medicine by the service pharmacy. A small stock of miltefosine was centralized at the Montes Claros health macro-region pharmacy, starting in October 2021, considering the large number of cases and requests generated in that region.

### Indicators of miltefosine access and use

In the first 36 months of the new treatment being made available, 286 requests for miltefosine were received, of which 281 were considered valid. After analyzing appropriateness of use, based on the criteria defined in Minas Gerais, 274 requests were granted and 7 had an unfavorable opinion for use. This was due to the lack of laboratory confirmation of the diagnosis of cutaneous leishmaniasis (4 requests), reported diagnosis of visceral leishmaniasis (1 request) and because 2 requests were for extending treatment for a period beyond the recommended 28 days. The request authorization rate was 97.51%. Among the authorized requests, 1 referred to retreatment of the same service user 17 months after their first treatment. This totaled 273 individuals who received treatment for cutaneous leishmaniasis with miltefosine.

Among the users treated with miltefosine, the majority were male (67.5%), median age 60.2 years, ranging from 13-98 years. The majority presented the cutaneous form of the disease (77.7%) and were classified as new cases (60.1%). The rate of women of childbearing age among the total number of patients treated with miltefosine was 5.5% (Table 1).

**Table 1:** Sociodemographic data of service users diagnosed as having cutaneous treated with miltefosine. Minas Gerais, 2021-2024 (n=273)

Variable	n (%)
Sex	
Male	184 (67.4)
Female	89 (32.6)
**Age** (years)	
>12	0 (0)
12-18	7 (2.6)
19-59	109 (39.9)
60+	156 (57.1)
Not reported	1 (0.4)
Schooling	
Illiterate	14 (5.2)
Incomplete elementary education	61 (22.4)
Complete elementary education	23 (8.5)
Complete high school education	40 (14.7)
Complete higher education	18 (6.6)
Not reported	116 (42.6)
**Race/skin color**	
White	68 (24.9)
Black	28 (10.3)
Asian	7 (2.6)
Mixed race	114 (41.8)
Indigenous	1 (0.4)
Not reported	54 (19.8)
**Forms of the disease**	
Cutaneous	212 (77.7)
Mucosal	60 (22.3)
**Types of case**	
New case	164 (60.1)
Recurrence	108 (39.9)
**Childbearing potential**	
At risk	15 (5.5)
Not at risk	259 (94.5)
**Residence** (**health macro**-region)	
Centro	84 (30.7)
Centro-Sul	10 (3.7)
Jequitinhonha	6 (2.2)
Leste	1 (0.4)
Leste do Sul	12 (4.4)
Nordeste	8 (2.9)
Noroeste	13 (4.8)
Norte	92 (33.7)
Oeste	6 (2.2)
Sudeste	1 (0.4)
Sul	29 (10.6)
Triângulo do Sul	2 (0.7)
Vale do Aço	9 (3.3)

**Figure 1:**
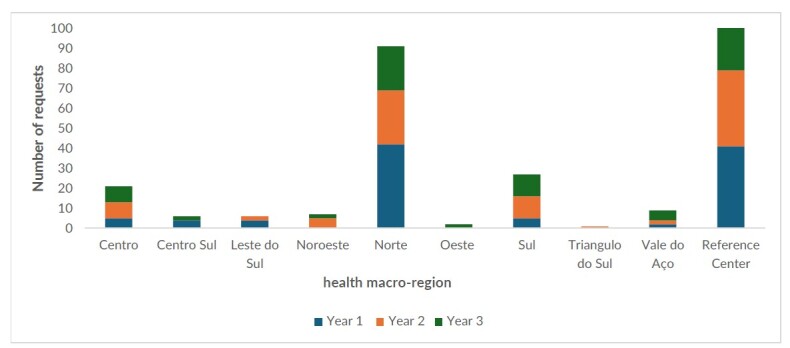
Miltefosine requests per Minas Gerais health macro-region and at the Reference Center, 2021-2024

The 274 authorized requests for use of miltefosine came from 59 health services, located in 42 cities in Minas Gerais, belonging to 9 of the state’s health macro-regions. The number of requests in the 3 subsequent 12-month periods were 101, 94 and 79 (Figure 1). The number of requests varied from one request in the Triângulo do Sul macro-region (0.4%) to 124 (45.6%) in the Centro macro-region, where the Reference Center is located. The Reference Center was the service that generated the most requests (n=103), responsible for 76.9% of the requests in the Centro macro-region.

Median time for analyzing requests was 1 day, with a range of 0-76 days. Among the macro-regions with at least 5 requests, Vale do Aço and Centro-Sul recorded the highest medians for the time taken to analyze requests, both 8 days. Median time between the request and effective distribution to the user’s reference pharmacy was 10 (0-340) days. The Reference Center was the health unit with the lowest median time, 0 (0-29) days for access to the medicine due to the service’s immediate dispensing flow. In the other services, median time taken to access miltefosine was 21.0 (0-340) days. Among the macro-regions with at least 5 requests, the Centro and Centro-Sul macro-regions had the lowest median time, 14.0 (0-87) and 16.5 (8-48) days. The Noroeste and Vale do Aço macro-regions had the largest time intervals, 32 (9-134) and 30 (17-66) days (Table 2).

**Table 2:** Time in days for analysis of requests for access to miltefosine. Minas Gerais, 2021-2024

Health macro-region (service)	Request analysis	Access (dispensing)	From diagnosis to dispensing
	n; median (lower-upper)		
Centro	21; 1 (0-12)	19; 14 (0-87)	19; 50 (13-299)
Centro-Sul	5; 8 (1-70)	5; 22 (8-48)	5; 90 (29-561)
Leste do Sul	5; 1 (0-4)	5; 22 (11-205)	5; 37 (30-660)
Noroeste	8; 0.5 (0-49)	5; 32 (9-134)	5; 177 (92-405)
Norte	91; 1 (0-76)	71; 21 (3-340)	71; 83 (14-1615)
Oeste	2; 2.5 (0-5)	2; 9.5 (9-10)	2; 67.5 (64.5-71)
Sul	27; 5 (0-30)	19; 25 (10-221)	19; 74 (22-241)
Triângulo do Sul	1; 6	1; 30	Not reported
Vale do Aço	8; 8 (0-23)	7; 30 (17-66)	7; 134 (84-986)
Reference Center	Not applicable	103; 0 (0-29)	103; 46 (0-3080)
Total	268; 0 (0-76)	237; 10 (0-340)	135; 43 (2-896)

**Figure 2:**
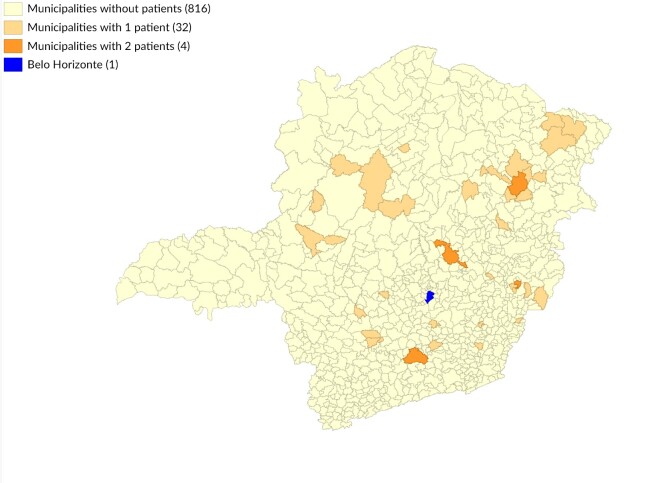
Municipality of residence of service users referred to the Reference Center treated with miltefosine. Minas Gerais, 2021-2024 (n=41)

In the case of 164 service users (60.0% of requests), the requests were for new cases of the disease in which miltefosine was considered the first therapeutic option. Median time between diagnosis of new cases and distribution of the drug was 43 (2-896) days, with the lowest median being observed at the Reference Center (2-79 days). In 119 cases, the request for miltefosine arose from a health center located in a municipality other than the user’s place of residence. Of these, 49 cases traveled to cities belonging to another health macro-region, traveling a median of 291.9 (14.8-719.8) km. Of the 103 service users who underwent treatment at the Reference Center, 41 lived in another health macro-region, traveling a median of 291.9 (21.3-719.8) km. The majority of those referred to the Reference Center came from the Nordeste (19.5%), Jequitinhonha (14.6%) and Vale do Aço (14.6%) macro-regions (Figure 2).

Among the authorized requests, 86.5% had the first stage of medicine dispensing recorded on the electronic system. Of these, 28 service users received full treatment the first time the medicine was dispensed, resulting in a single record for their entire treatment. Among the macro-regions with the highest number of requests, the Sul and Norte macro-regions had the lowest recording rates on the electronic system, namely 40.7% and 38.0%, respectively.

Based on this information, the degree of miltefosine diffusion was estimated at 11.3% (Table 3).

**Table 3:** Miltefosine diffusion by health macro-region. Minas Gerais, 2021-2024

Health macro-regions (residence)	Confirmed cutaneous leishmaniasis cases per place of residence	Miltefosine users per place of residence	Diffusion (%)
Sul	31	28	90.3
Centro-Sul	45	10	22.2
Centro	332	84	25.3
Jequitinhonha	152	6	3.9
Oeste	68	6	8.8
Leste	45	1	2.2
Sudeste	47	1	2.1
Norte	888	92	10.4
Noroeste	105	13	12.3
Leste do Sul	193	12	6.2
Nordeste	225	9	4.0
Triângulo do Sul	9	2	22.2
Triângulo do Norte	14	0	0.0
Vale do Aço	227	9	3.9
Extremo sul	30	0	0.0
Sudoeste	4	0	0.0
Total	2.415	273	11.3

## Discussion

For the first three years of miltefosine implementation in Minas Gerais, we found a low degree of diffusion of this technology and heterogeneity in access to it between the health macro-regions. The Reference Center concentrated most of the requests, had the shortest time to access treatment and received referrals from many service users from regions with a low number or no requests for the drug during this period.

This study has several limitations related to potential sources of bias or inaccuracy contained in administrative databases. These limitations are related to lack of data quality control, underreporting or incomplete information, linked to possible barriers such as unavailability of trained professionals, infrastructure, or logistical issues. The study was not able to capture contextual information such as qualitative aspects of the experience of service users and health professionals, satisfaction, difficulties or perception of treatment. It should be noted that this descriptive analysis provides information up to access to medicine, while information on its use and outcomes needs to be supplemented in future studies by composing new outcome indicators. The findings of this analysis have great potential for guiding new processes and indicators that favor access to technologies in the SUS.

The process of incorporating miltefosine that took place in 2018 estimated an initial diffusion rate of 10.0% in the first year, with an annual increase of 10.0%, predicting that it would reach 50.0% of cutaneous leishmaniasis cases in five years, or up to 90.0% in a more aggressive diffusion scenario (18). The results indicate that this projection was overestimated, since the diffusion rate of 11.0% after three years of implementation in a state where leishmaniasis is endemic confirms that there are several factors to be investigated. The fact of miltefosine only becoming available in 2021 reflected complementary actions to ensure its safe use by the population related to legislation and flows necessary for access to it (7,14).

Implementation of new practices or technologies and adherence to them are measured by frameworks that assess adoption, coverage, and acceptability of technologies when fully available (19,20). In cases of shortages, lack of access becomes an almost insurmountable barrier, as observed with miltefosine, the supply of which, restricted to the strategic component of pharmaceutical assistance, compromises all planning and implementation strategies outlined for its use in clinical practice. Two moments of shortages in the network compromised access to miltefosine, increasing the time for dispensing the drug, including at the Reference Center, the only service that dispensed the drug immediately.

Miltefosine implementation required coordination of several processes, including a rigorous flow of requests and checks to ensure safe use of the drug. This was particularly relevant for women at risk of pregnancy, who represented 5.5% of miltefosine users during this period. Use of teratogenic drugs, such as miltefosine, by women of childbearing potential is a significant barrier to large-scale implementation, as it requires strict monitoring and effective adherence to contraceptive methods, which can generate resistance among both health professionals and service users. The experience with thalidomide in the leprosy program illustrates the challenges and weaknesses of the surveillance system and reinforces the need for improvements with the introduction of miltefosine in the SUS (21). The profile of adverse events, although mostly mild and related to the gastrointestinal tract (22), requires clinical monitoring of renal function, this being a previously identified need (23).

Recent warnings from health authorities about possible reproductive (24) and ocular (25) events reinforce the need for greater vigilance. Such issues will need to be investigated and complemented with acceptability studies, which consider different perspectives and are essential for successful implementation (26).

The agility in the analysis and the high authorization rate of miltefosine requests in Minas Gerais may reflect the structure organized to support implementation and effective communication between the parties involved. This result may be masked by preliminary analyses of many requests that were returned to the applicants and were only recorded after all the necessary documents had been gathered. This length of time was not accounted for in the formal analysis, but certainly impacts the start of treatment for users. A parallel project, between 2022 and 2023, improved access and quality indicators in the miltefosine implementation process in Minas Gerais by applying the Joanna Briggs Institute (JBI) evidence implementation framework (27). This project used several communication strategies aimed at health professionals in the health macro-regions and may have been essential in correcting problems detected in the second year of monitoring, impacting the indicators measured.

The Reference Center, with its differentiated flow and immediate dispensing, is a demonstration of an independent model with the potential to improve access to miltefosine. It is worth noting that this service has a robust structure for surveillance and monitoring, something not replicated in many services that face difficulties in the provision of consultations and laboratory tests. This represents a barrier to decentralization of access, especially for the most vulnerable populations (28).

Discrepancies identified in miltefosine diffusion between regions suggested the need for specific approaches. The Norte region, which concentrated the largest number of cases of cutaneous leishmaniasis, had low miltefosine diffusion and the longest time between diagnosis and dispensing, despite maintaining a small stock of the drug in the regional pharmacy. The Sul macro-region showed high diffusion, but with a longer time of access to miltefosine. Both regions had the lowest rates of dispensing records input on the system, which compromised surveillance and traceability of the use of miltefosine. All these aspects should be discussed in order to establish the most effective access model and component of pharmaceutical assistance for provision of miltefosine (29).

Large time ranges for access indicators highlight, in addition to data heterogeneity, possible local barriers. The large variability in miltefosine request time may reflect the ability to organize multiple documents, related to local work flows, access to information by health professionals and agility of health teams. The time taken to access miltefosine may reflect issues regarding the provision of health services (consultations and laboratory tests) and miltefosine itself, which is largely impacted when there are shortages. Mapping the locations with the best performance becomes a sequential action for proposing strategies to improve miltefosine implementation and dissemination in the years to come.

Eighteen percent of users had to travel to other macro-regions, traveling an average of more than 300 km to receive treatment, which increased access time and associated costs. Regional organization and lack of knowledge about the work flow needed in order to obtain miltefosine may explain this movement, generating inequalities in access to treatment (30). Analysis of miltefosine prescriptions at the Reference Center for people from macro-regions with a low number or absence of miltefosine requests may help map the most deficient access routes in the state, thus improving miltefosine implementation in the years to come.

Miltefosine implementation in Minas Gerais has revealed challenges such as variations in distribution and dispensing times between regions and gaps in records that compromise drug surveillance and traceability. These obstacles reflect the complexity of introducing new technologies into the SUS, highlighting the importance of continuous improvement strategies to ensure that more people have rapid, equitable and safe access to available treatments.

## Data Availability

The database and the analysis codes used in this research are available at: https://data.mendeley.com/preview/fbbk4r3tz4?a=e9063b49-fa79-43e6-820a-4916e04ef514 (31).
