# Supplementary material for: Miltefosine implementation for treatment of cutaneous leishmaniasis: access indicators in the state of Minas Gerais, 2021-2024
Source: Epidemiol Serv Saude. 2025 Jun 20;34:e20240690. doi: 10.1590/S2237-96222025v34e20240690.en (PMC12204067; doi:10.1590/S2237-96222025v34e20240690.en)
Supplement: Translation

# Translation: Implementação da miltefosina no tratamento de leishmaniose tegumentar: indicadores de acesso em Minas Gerais, 2021-2024

**DOI:** 10.1590/S2237-96222025v34e20240690.pt

**Authors:** Sarah Nascimento Silva, Mell Ferreira Saliba, Laís Raquel Ribeiro, Gláucia Cota

**Affiliations:** 1 Núcleo de Avaliação de Tecnologias em Saúde, Instituto René Rachou, Fundação Oswaldo CruzBelo Horizonte, MG, Brazil

**Keywords:** Leishmaniose, Acesso a Medicamentos Essenciais e Tecnologias em Saúde, Ciência da Implementação, Sistema Único de Saúde, Estudos Transversais

## Abstract

**Objetivo:**

Descrever e avaliar os primeiros indicadores da implementação-piloto da miltefosina em Minas Gerais para o tratamento da leishmaniose tegumentar.

**Métodos:**

Trata-se de estudo descritivo e transversal com base em documentos normativos e dados secundários administrativos, gerados durante a dispensação de miltefosina em Minas Gerais entre maio de 2021 e abril de 2024. Foram calculados os indicadores relacionados ao acesso e à operacionalidade da estratégia implementada para as etapas envolvidas na aprovação da solicitação de uso e dispensação do medicamento.

**Resultados:**

A taxa de deferimento foi 97,5% para 281 solicitações válidas. O ambulatório do Instituto René Rachou, centro de referência em leishmaniose para Minas Gerais, foi responsável pelo maior número de solicitações no período. As macrorregionais de saúde Centro e Norte concentraram o maior número de solicitações (48,9% e 33,6%), o que coincidiu com as regiões com maior número de casos notificados. As medianas de tempo para a análise das solicitações e o acesso da pessoa ao medicamento foram 1,0 (0-76) e 10,0 (0-340) dias, com grande variação entre as macrorregionais de saúde. Houve 119 pessoas atendidas em município diverso de sua residência. Destas, 49 precisaram se deslocar para outra macrorregional de saúde. A taxa de difusão do medicamento foi 11,3% para os três anos de monitoramento, com grande variação entre as macrorregionais.

**Conclusão:**

Os indicadores confirmaram a implementação da miltefosina, mas revelaram baixa difusão e disparidades regionais. Assim é necessário identificar barreiras e traçar estratégias para ampliar o acesso equânime às novas tecnologias.

**Aspectos éticos**

A presente pesquisa respeitou os princípios éticos, obtendo os seguintes dados de aprovação: :

Comitê de ética em pesquisa: Instituto René Rachou

Número do parecer: 5.238.337

Data de aprovação: 20/12/2021

Certificado de apresentação de apreciação ética: 45481421.4.0000.5091

Registro do consentimento livre e esclarecido: Obtido de todos os participantes antes da coleta.

## Introdução

A leishmaniose tegumentar é uma doença infecciosa, não contagiosa, causada por várias espécies do gênero Leishmania e transmitida pela picada de mosquito predominante em regiões tropicais (1). Com distribuição global ampla, afeta principalmente populações negligenciadas em áreas subdesenvolvidas, o que acarreta considerável morbidade e impacto socioeconômico (2). O Brasil estava entre os oito países responsáveis por 85,0% dos casos mundiais com média anual de 16 mil casos registrados entre 2018 e 2022 (3). A doença representa um desafio sob a perspectiva de política pública, pois exige esforço técnico, operativo e político. Há dificuldade de intervenção no ciclo de transmissão que é associado à interação ambiental e à ampla distribuição geográfica nas Américas (1,4).

O acesso ao tratamento da leishmaniose tegumentar é uma das estratégias de controle da doença, mas limitado pelas dificuldades no diagnóstico e pelo reduzido número de opções terapêuticas, que incluía tratamentos parenterais, administrados por tempo prolongado e associados à considerável toxicidade (1). A miltefosina é o primeiro medicamento oral para o tratamento da leishmaniose visceral e tegumentar, cuja disponibilização representa avanço significativo em termos de descentralização do acesso por permitir a autoadministração no domicílio (5,6). O medicamento foi incorporado ao Sistema Único de Saúde (SUS) em 2018 e, após um longo processo de regulamentação (7) e aquisição internacional, começou a ser disponibilizado pelo Ministério da Saúde às secretarias estaduais em 2021, por meio do componente Estratégico da Assistência Farmacêutica (8). O potencial de teratogenicidade da miltefosina exigiu rigoroso controle do uso e equipe preparada para monitorar os usuários, com foco especial nas mulheres em idade fértil (9).

A implementação de medicamento recém-incorporado ao SUS agrega diversos desafios que transcendem a disponibilização no serviço. A superação desses desafios exige que se compreenda os entraves à sua oferta (10), a resistência ou o desconhecimento de sua disponibilidade e a adoção de estratégias que consolidem o seu uso na prática clínica dos serviços. Embora os processos de incorporação estejam bem definidos no SUS (11), os processos de implementação apresentam grades lacunas para os gestores em termos de diretrizes e orientações federais para sua execução (12). Indicadores relacionados ao alcance dos medicamentos no sistema de saúde constituem dados fundamentais para compreender a capilaridade com que a disponibilização foi realizada, um dos fatores essenciais para a implementação efetiva (13). 

A partir da distribuição da miltefosina pelo Ministério da Saúde, cada estado teve autonomia para definir sua estratégia de dispensação (14). Em Minas Gerais, região endêmica para leishmaniose tegumentar, a secretaria estadual de saúde firmou a parceria de apoio técnico com o Centro de Referência em Leishmaniose do Instituto René Rachou para a concepção e execução do plano de implementação-piloto da miltefosina (15). Essa experiência representou a oportunidade para o desenvolvimento de estratégia sistematizada para orientar e monitorar a implementação de tecnologias em saúde no Brasil. Este estudo teve como objetivo descrever e avaliar os primeiros indicadores da estratégia de implementação-piloto da miltefosina em Minas Gerais.

## Métodos

### Delineamento e contexto

Trata-se de estudo descritivo e transversal com base em fontes secundárias de informação. Este descreve a experiência da implementação-piloto da miltefosina em Minas Gerais e a análise de indicadores de acesso, entre maio de 2021 e abril de 2024, totalizando 36 meses. 

### Participantes e variáveis 

Os participantes compreendem usuários do SUS com o diagnóstico de leishmaniose tegumentar e a indicação de tratamento com a miltefosina. Foram analisadas as variáveis sociodemográficas sexo (feminino, masculino), idade, local de residência (município), escolaridade (analfabetos, ensino fundamental, médio, superior) e raça/cor da pele. Analisaram-se as variáveis clínicas sobre a forma da doença (cutânea, mucosa), tipo de caso (novo, recidiva), data do diagnóstico e potencial de engravidar (com risco, sem risco). Foram descritas variáveis relacionadas aos processos de solicitação do medicamento pelas variáveis local de residência e local do serviço (município e macrorregional de saúde de Minas Gerais definida conforme o plano diretor de regionalização do estado), distância percorrida, data de solicitação, data de emissão de parecer e datas da dispensação e do registro em sistema. As medidas a seguir foram escolhidas como indicadores de acesso.

1. **Taxa de deferimento das solicitações do medicamento**: razão entre o número de solicitações aprovadas em relação ao número total de solicitações protocoladas na secretaria estadual de saúde que foram consideradas válidas (excluídas duplicatas), multiplicado por 100.
2. **Taxa de mulheres em idade fértil em uso da miltefosina**: razão entre o número de mulheres em idade fértil e o número total de pessoas tratadas com miltefosina, multiplicado por 100. Definiram-se mulheres em idade fértil aquelas entre 12 e 55 anos, excluídas as submetidas a procedimento de esterilização definitiva, ou mulheres em menopausa confirmada há, no mínimo, dois anos.
3. **Tempo de análise das solicitações**: intervalo em dias entre a data na qual o e-mail com a solicitação de miltefosina foi recebido na secretaria estadual de saúde (protocolo) até a data de emissão do parecer final. O serviço de referência é excluído dessa análise, pois não apresenta esta etapa.
4. **Tempo para acesso ao medicamento**: intervalo em dias entre a data na qual o e-mail com a solicitação de miltefosina foi recebido na secretaria estadual de saúde (protocolo) até a data em que o medicamento foi dispensado ao usuário na farmácia designada.
5. **Tempo entre o diagnóstico e a dispensação do medicamento**: intervalo em dias entre a data do diagnóstico de leishmaniose tegumentar e o dia em que o medicamento foi dispensado ao usuário. Calculada apenas para casos novos da doença, reflete o prazo completo para acesso ao tratamento após a confirmação do diagnóstico de leishmaniose tegumentar.
6. **Taxa de registro da dispensação** (**primeira etapa**): razão entre o número de dispensações da miltefosina registradas no sistema de dispensação e o número de autorizações de dispensação do medicamento realizadas pela secretaria estadual de saúde na primeira etapa de dispensação do medicamento, multiplicado por 100. Reflete a adequação do processo de registro de dispensação, visto que os lançamentos no sistema de dispensação são obrigatórios para a miltefosina.
7. **Grau de difusão**: razão entre o número de solicitações aprovadas do medicamento e o número total de casos de leishmaniose tegumentar diagnosticados no estado para o período divulgado na plataforma TabNet saúde até 9 de setembro de 2024. Representa medida de penetração, ou seja, a integração da intervenção na prática.

### Fontes de dados e mensuração 

Dados administrativos de documentos atrelados à solicitação e prescrição de miltefosina constituem fonte de dados do estudo: prescrição, laudo do exame diagnóstico, relatório médico, formulário de solicitação e notificação de leishmaniose tegumentar e o formulário do Sistema de Informação de Agravos de Notificação. Foram analisados também outros documentos gerados no âmbito do componente estratégico da assistência farmacêutica nesse período, relacionados ao gerenciamento da solicitação da miltefosina, tais como planilhas com as solicitações, e-mails de comunicação para análise dos pedidos e o registro de contabilidade do medicamento a partir dos relatórios do Sistema Integrado de Gerenciamento da Assistência Farmacêutica. 

### Controle de viés e tamanho do estudo 

Os dados de solicitação do medicamento foram compilados em um banco pareando informações sobre a solicitação do medicamento, a resposta de autorização ou recusa e as datas de dispensação. A amostra deste estudo compreendeu todos os registros de solicitação gerados no período analisado. Após o processo de limpeza do banco pela retirada de duplicatas, foi realizada a pseudoanonimização da base principal, por dois pesquisadores independentes, gerando uma nova base de dados para realização das análises. 

### Métodos estatísticos 

A população que teve acesso ao medicamento foi caracterizada a partir da descrição de medidas de tendência central e dispersão (mínimo-máximo) para as variáveis sociodemográficas e clínicas usando o software Statistical Package for the Social Sciences versão 23. Foram calculadas medidas relacionadas ao acesso e à operacionalidade da estratégia de implementação da miltefosina conforme os indicadores definidos. Os resultados foram apresentados de forma global e estratificada pelas macrorregionais de saúde. A análise sobre o deslocamento foi realizada para todos os usuários identificando a distância percorrida entre o local de residência e o serviço que realizou o atendimento. No serviço com maior número de usuários externos, foi realizado o georreferenciamento dos municípios no mapa de Minas Gerais a partir da localização dos municípios de residência, utilizando o software GeoDa versão1.22.0.4. Este estudo adotou as checklists Reporting of studies Conducted using Observational Routinely collected health Data (16) e Strengthening the Reporting of Observational Studies in Epidemiology (17).

## Resultados

### Descrição da estratégia de implementação

A estratégia de implementação adotada foi estruturada em etapas tendo como inspiração a ferramenta de gestão e controle de processo Ciclo de Deming, com as etapas Plan, Do, Check e Act. Estas foram adaptadas de forma a compor quatro conjunto de ações designadas “mapear, preparar, pilotar e monitorar” dando origem à abordagem genérica para orientar implementação em saúde, que foi chamada de ImplementaSUS. 

Após análise do contexto em que se daria o uso da nova tecnologia e das exigências já estabelecidas para seu uso, foram definidos o fluxo de aprovação de uso e distribuição a ser adotado no estado, o conjunto de documentos mínimos que deveriam subsidiar a prescrição do tratamento com miltefosina e as ações de monitoramento desses processos. O centro de referência adotou fluxo diferenciado com emissão do parecer de aprovação interno e dispensação imediata do medicamento pela farmácia do serviço. A centralização de pequeno estoque de miltefosina foi realizado na farmácia da macrorregional de saúde de Montes Claros, a partir de outubro de 2021, considerando o grande número de casos e solicitações gerados nessa regional.

### Indicadores de acesso e uso da miltefosina 

Nos primeiros 36 meses de disponibilização do novo tratamento, foram recebidas 286 solicitações de miltefosina, das quais 281 foram consideradas válidas. Após análise da pertinência de uso, com base nos critérios definidos em Minas Gerais, 274 pedidos foram deferidos e 7 tiveram parecer não favorável ao uso. Isso se deu pela inexistência de confirmação laboratorial do diagnóstico de leishmaniose tegumentar (4 solicitações), pelo diagnóstico informado para leishmaniose visceral (1 solicitação) e por, em 2 pedidos, tratar-se de solicitação de extensão do tratamento por período além dos 28 dias preconizados. A taxa de deferimento das solicitações foi 97,51%. Entre as solicitações deferidas, 1 correspondeu ao retratamento do mesmo usuário após 17 meses do primeiro tratamento. Isso totalizou 273 indivíduos que receberam o tratamento de leishmaniose tegumentar com miltefosina. 

Entre os usuários tratados com miltefosina, a maior parte era do sexo masculino (67,5%), idade mediana 60,2 anos, variando de 13-98 anos. A maioria apresentava a forma cutânea da doença (77,7%) e era classificada como caso novo (60,1%). A taxa de mulheres em idade fértil entre o total de tratados com miltefosina foi 5,5% (Tabela 1). 

**Tabela 1:** Dados sociodemográficos dos usuários diagnosticados com leishmaniose tegumentar tratadas com miltefosina. Minas Gerais, 2021-2024 (n=273)

Variável	n (%)
**Sexo**	
Masculino	184 (67,4)
Feminino	89 (32,6)
**Idade** (anos)	
>12	0 (0)
12-18	7 (2,6)
19-59	109 (39,9)
60+	156 (57,1)
Não relatado	1 (0,4)
Escolaridade	
Analfabetos	14 (5,2)
Ensino fundamental incompleto	61 (22,4)
Ensino fundamental completo	23 (8,5)
Ensino médio completo	40 (14,7)
Ensino superior completo	18 (6,6)
Não relatado	116 (42,6)
**Raça/cor da pele**	
Brancos	68 (24,9)
Pretos	28 (10,3)
Amarelos	7 (2,6)
Pardos	114 (41,8)
Indígenas	1 (0,4)
Não relatado	54 (19,8)
**Formas da doença**	
Cutânea	212 (77,7)
Mucosa	60 (22,3)
**Tipos de caso**	
Caso novo	164 (60,1)
Recidiva	108 (39,9)
**Potencial de engravidar**	
Com risco	15 (5,5)
Sem risco	259 (94,5)
**Residência** (**macrorregional de saúde**)	
Centro	84 (30,7)
Centro-Sul	10 (3,7)
Jequitinhonha	6 (2,2)
Leste	1 (0,4)
Leste do Sul	12 (4,4)
Nordeste	8 (2,9)
Noroeste	13 (4,8)
Norte	92 (33,7)
Oeste	6 (2,2)
Sudeste	1 (0,4)
Sul	29 (10,6)
Triângulo do Sul	2 (0,7)
Vale do Aço	9 (3,3)

As 274 solicitações deferidas para o uso da miltefosina eram procedentes de 59 serviços de saúde, situados em 42 cidades mineiras, pertencentes a 9 macrorregionais de saúde do estado. O número de solicitações nos 3 períodos subsequentes de 12 meses foram 101, 94 e 79 (Figura 1). O número de solicitações variou de um pedido na macrorregional Triângulo do Sul (0,4%) até 124 (45,6%) na macrorregional Centro, onde se localiza o centro de referência. O centro de referência foi o serviço que gerou mais solicitações, (n=103), responsável por 76,9% das solicitações da macrorregional Centro.

**Figura 1:** Solicitações de miltefosina por macrorregional de saúde de Minas Gerais e no centro de referência, 2021-2024

A mediana de tempo para análise das solicitações foi de 1 dia, com variação de 0-76 dias. Entre as macrorregionais com pelo menos 5 solicitações, Vale do Aço e Centro-Sul registraram as maiores medianas para o tempo de análise das solicitações, em ambas de 8 dias. A mediana de tempo entre a solicitação e a efetiva distribuição à farmácia de referência do usuário foi 10 (0-340) dias. O centro de referência foi a unidade de saúde com a menor mediana de tempo, 0 (0-29) dia para o acesso ao medicamento em função do fluxo de dispensação imediata pelo serviço. Nos demais serviços, a mediana de tempo para acesso à miltefosina foi 21,0 (0-340) dias. Entre as macrorregionais com pelo menos 5 solicitações, as macrorregionais Centro e Centro-Sul apresentaram as menores mediana de tempo, 14,0 (0-87) e 16,5 (8-48) dias. Noroeste e Vale do Aço apresentaram os maiores intervalos de tempo, 32 (9-134) e 30 (17-66) dias (Tabela 2). 

**Tabela 2:** Tempo em dias para análise das solicitações e acesso à miltefosina. Minas Gerais, 2021-2024

Macrorregional de saúde (serviço)	Análise da solicitação	Acesso (dispensação)	Diagnóstico até a dispensação
	n; mediana (mínimo-máximo)		
Centro	21; 1 (0-12)	19; 14 (0-87)	19; 50 (13-299)
Centro-Sul	5; 8 (1-70)	5; 22 (8-48)	5; 90 (29-561)
Leste do Sul	5; 1 (0-4)	5; 22 (11-205)	5; 37 (30-660)
Noroeste	8; 0,5 (0-49)	5; 32 (9-134)	5; 177 (92-405)
Norte	91; 1 (0-76)	71; 21 (3-340)	71; 83 (14-1615)
Oeste	2; 2,5 (0-5)	2; 9,5 (9-10)	2; 67,5 (64,5-71)
Sul	27; 5 (0-30)	19; 25 (10-221)	19; 74 (22-241)
Triângulo do Sul	1; 6	1; 30	Não relatado
Vale do Aço	8; 8 (0-23)	7; 30 (17-66)	7; 134 (84-986)
Centro de referência	Não se aplica	103; 0 (0-29)	103; 46 (0-3080)
**Total**	268; 0 (0-76)	237; 10 (0-340)	135; 43 (2-896)

Em 164 usuários (60,0% das solicitações), as solicitações se tratavam de casos novos da doença em que a miltefosina foi considerada a primeira opção terapêutica. A mediana de tempo entre o diagnóstico dos casos novos até a distribuição do medicamento foi 43 (2-896) dias, sendo observada a menor mediana no centro de referência (2-79 dias). Em 119 casos, a solicitação de miltefosina foi deflagrada em unidade de saúde localizada em município diverso daquele de residência do usuário. Desses, 49 se deslocavam para cidades que pertencem a outra macrorregional de saúde, percorrendo a mediana de 291,9 (14,8-719,8) km. Dos 103 usuários que realizaram tratamento no centro de referência, 41 residiam em outra macrorregional de saúde, percorrendo a mediana de 291,9 (21,3 – 719,8) km. A maior parte encaminhada ao centro de referência era procedente das macrorregionais Nordeste (19,5%), Jequitinhonha (14,6%) e Vale do Aço (14,6%) (Figura 2).

**Figura 2:** Município de residência de usuários encaminhados ao centro de referência tratados com miltefosina. Minas Gerais, 2021-2024 (n=41)

Entre as solicitações aprovadas, 86,5% tiveram registro da primeira etapa da dispensação do medicamento no sistema eletrônico. Destas, 28 usuários receberam o tratamento total já na primeira dispensação, resultando em um único registro para todo o tratamento. Entre as macrorregionais com maior número de solicitações, Sul e Norte apresentaram as menores taxas de registro no sistema eletrônico, 40,7% e 38,0%. O número de casos de leishmaniose tegumentar notificados em Minas Gerais, no mesmo período desta análise, foi 2.415. Com base nessa informação, estimou-se o grau de difusão da miltefosina em 11,3% (Tabela 3). 

**Tabela 3:** Difusão da miltefosina por macrorregiões de saúde. Minas Gerais, 2021-2024

Macrorregionais de saúde (residência)	Casos confirmados de leishmaniose tegumentar por local de residência	Usuários da miltefosina por local de residência	Difusão (%)
Sul	31	28	90,3
Centro-Sul	45	10	22,2
Centro	332	84	25,3
Jequitinhonha	152	6	3,9
Oeste	68	6	8,8
Leste	45	1	2,2
Sudeste	47	1	2,1
Norte	888	92	10,4
Noroeste	105	13	12,3
Leste do Sul	193	12	6,2
Nordeste	225	9	4,0
Triângulo do Sul	9	2	22,2
Triângulo do Norte	14	0	0,0
Vale do Aço	227	9	3,9
Extremo sul	30	0	0,0
Sudoeste	4	0	0,0
Total	2.415	273	11,3

## Discussão

Nos primeiros três anos de implementação da miltefosina em Minas Gerais, observou-se baixo grau de difusão da tecnologia e heterogeneidade no acesso à miltefosina entre as macrorregionais de saúde. O centro de referência concentrou a maior parte das solicitações, apresentou o menor tempo de acesso ao tratamento e recebeu o encaminhamento de muitos usuários de regiões com número baixo ou ausência de solicitações do medicamento nesse período. 

O estudo apresenta diversas limitações atreladas às potenciais fontes de viés ou imprecisão provenientes de bases de dados administrativas. Essas limitações estão relacionadas à ausência de controle da qualidade dos dados, subnotificações ou incompletude das informações, atrelada a possíveis barreiras como indisponibilidade de profissionais capacitados, infraestrutura, ou questões logísticas. O estudo não foi capaz de capturar informações do contexto como aspectos qualitativos da experiência dos usuários e profissionais, satisfação, dificuldades ou percepção do tratamento. Destaca-se que esta análise descritiva dispõe de informações até o acesso aos medicamentos, informações sobre o uso e desfechos precisam ser complementadas em estudos futuros compondo novos indicadores de resultados. Os achados desta análise têm grande potencial para orientar novos processos e indicadores que favoreçam o acesso a tecnologias no SUS.

O processo de incorporação da miltefosina realizado em 2018 dimensionou a difusão inicial de 10,0% no primeiro ano, com acréscimo anual de 10,0%, prevendo o alcance de 50,0% dos casos de leishmaniose tegumentar em cinco anos, ou até 90,0% em um cenário de difusão mais agressivo (18). Os resultados apontam que essa projeção foi superestimada, pois a difusão de 11,0% após três anos de implementação em um estado endêmico para a doença confirma que há vários fatores a serem apurados. A disponibilização da miltefosina apenas em 2021 refletiu as ações complementares para garantir o uso seguro da miltefosina pela população relacionada a legislação e fluxos necessários para acesso ao medicamento (7,14).

A implementação e a adesão a novas práticas ou tecnologias são mensuradas em estruturas que avaliam a adoção, cobertura e aceitabilidade das tecnologias quando totalmente disponíveis (19,20). Em casos de desabastecimento, a falta de acesso torna-se uma barreira quase intransponível, como observado com a miltefosina, cujo fornecimento restrito ao componente estratégico compromete todo o planejamento e as estratégias de implementação delineadas para seu uso na prática clínica. Dois momentos de desabastecimento da rede comprometeram o acesso à miltefosina, aumentando o tempo para a dispensação do medicamento, inclusive no centro de referência, único serviço que realizava a dispensação imediata do medicamento. 

A implementação da miltefosina exigiu a coordenação de diversos processos, incluindo um fluxo rigoroso de requisições e conferências para garantir o uso seguro do medicamento. Isso foi particularmente relevante para mulheres sob risco de gravidez, que representaram 5,5% dos usuários da miltefosina nesse período. A utilização de medicamentos teratogênicos, como a miltefosina, por mulheres em idade fértil é uma barreira significativa à implementação em larga escala, pois exige monitoramento rigoroso e adesão eficaz aos métodos contraceptivos, o que pode gerar resistência tanto entre profissionais quanto entre usuários. A experiência com a talidomida no programa de hanseníase ilustra os desafios e as fragilidades do sistema de vigilância e reforça a necessidade de aprimoramentos com a introdução da miltefosina no SUS (21). O perfil de eventos adversos, embora na maioria leves e relacionados ao trato gastrointestinal (22), exige monitoramento clínico da função renal, necessidade já identificada em outro estudo (23). 

Alertas recentes de autoridades sanitárias sobre possíveis eventos reprodutivos (24) e oculares (25) reforçam a necessidade de maior vigilância. Tais questões precisarão ser investigadas e complementadas com estudos de aceitabilidade, que consideram diversas perspectivas e são fundamentais para a implementação bem-sucedida (26).

A agilidade na análise e a alta taxa de deferimento das solicitações da miltefosina em Minas Gerais podem refletir a estrutura organizada de apoio à implementação e a comunicação eficaz entre as partes envolvidas. Esse resultado pode estar mascarado por análises preliminares de muitos pedidos que retornavam aos solicitantes e eram protocolados somente após reunir todos os documentos necessários, um tempo não contabilizado na análise formal, mas que certamente impacta no início do tratamento dos usuários. Um projeto paralelo, entre 2022 e 2023, melhorou indicadores de acesso e qualidade no processo de implementação da miltefosina no estado aplicando a estrutura da JBI (27). Esse projeto utilizou diversas estratégias de comunicação voltadas aos profissionais das macrorregionais de saúde e pode ter sido essencial para corrigir problemas detectados no segundo ano de monitoramento, impactando nos indicadores mensurados. 

O centro de referência, com o fluxo diferenciado e a dispensação imediata, demonstra modelo independente com potencial para melhorar o acesso à miltefosina. Vale ressaltar que esse serviço possui estrutura robusta para vigilância e monitoramento, algo não replicado em muitos serviços que enfrentam dificuldades para oferta de consultas e exames. Isso representa uma barreira à descentralização do acesso principalmente para as populações mais vulneráveis (28). 

Discrepâncias identificadas na difusão da miltefosina entre as regiões sugeriram a necessidade de abordagens específicas. A região Norte, que concentrava o maior número de casos de leishmaniose tegumentar, teve a difusão baixa do medicamento e o maior tempo entre diagnóstico e dispensação, apesar de manter um pequeno estoque do medicamento na farmácia regional. A macrorregião Sul apresentou alta difusão, mas com maior tempo de acesso ao medicamento. Em ambas as regiões, houve os menores índices de registro da dispensação em sistema, o que comprometeu a vigilância e a rastreabilidade do uso do medicamento. Todos esses aspectos devem ser discutidos para estabelecer o modelo de acesso e o componente da assistência farmacêutica mais efetivo para a oferta da miltefosina (29).

Grandes amplitudes de tempo para indicadores de acesso destacam, além da heterogeneidade dos dados, possíveis barreiras locais. A grande variabilidade de tempo na solicitação do medicamento pode refletir a capacidade de organização de múltiplos documentos, relacionada aos fluxos locais, o acesso à informação pelos profissionais e a agilidade das equipes. O tempo de acesso à miltefosina pode refletir questões sobre a oferta dos serviços de saúde (consultas e exames) e do medicamento, amplamente impactado por momentos de desabastecimento. O mapeamento das localidades com melhores desempenhos torna-se ação sequencial para proposição de estratégias para aprimorar a implementação e a difusão do medicamento nos próximos anos.

Dezoito por cento dos usuários precisaram se deslocar para outras macrorregionais, percorrendo, em média, mais de 300 km para receber tratamento, o que agravou o tempo de acesso e os custos associados. A organização regional e o desconhecimento dos fluxos para obtenção do medicamento podem explicar essa movimentação, gerando desigualdades no acesso ao tratamento (30). A análise das prescrições de miltefosina no centro de referência para pessoas procedentes de macrorregionais com número baixo ou ausência de solicitações da miltefosina pode auxiliar a mapear as rotas de acesso mais deficientes no estado, aprimorando a implementação do medicamento nos próximos anos.

A implementação da miltefosina em Minas Gerais revelou desafios como a variação na difusão e no tempo de dispensação entre regiões e lacunas nos registros que comprometem a vigilância e rastreabilidade do medicamento. Esses obstáculos refletem a complexidade da introdução de novas tecnologias no SUS, ressaltando a importância de estratégias contínuas de aprimoramento para assegurar que mais pessoas tenham acesso rápido, equânime e seguro aos tratamentos disponíveis.

## Data Availability

O banco de dados e os códigos de análise utilizados na pesquisa estão disponíveis em: https://data.mendeley.com/datasets/fbbk4r3tz4/1 (31).
